# Dr. Clark on the Influence of Climate in the Prevention and Cure of Diseases

**Published:** 1829-07-01

**Authors:** 


### XIII.

The Influence of Climate in the Prevention and Cure of Chronic Diseases, more particularly of the Chest and Digestive Organs: Comprising an Account of the Principal Places resorted to by Invalids in England and the South of Europe; a Comparative Estimate of their respective Merits in Particular Diseases; and General Directions for Invalids while travelling and residing Abroad. With an Appendix, containing a Series of Tables on Climate. By *James Clark*, M.D. &c. &c. 8vo. pp. 328. London. June, 1829.

The influence of climate on the moral and physical character of man, as well as on his health, has not ceased to attract the attention of philosophers and physicians from Hippocrates to Cabanis—or, (to bring things to the very level of the times) Dr. James Clark. The subject is exceedingly extensive. An able foreign writer, in an article on climate, tells us that, in a Hygienic point of view alone, the influence of climate embraces, "a consideration of all those alterations produced in the human body by the air we breathe, the waters we drink, the localities of our residence—the region of the earth, the seasons of the year, the prevailing winds, the temperature, the elevation or depression of surface, the dryness or humidity, the fertility or sterility of the soil—the food which that soil produces, &c." A treatise, so complete in all its parts, as to meet the views of M. Virey, (the author alluded to) is not to be expected from any one pen. It would require a work nearly as large as that of Dr. Good on the Study of Medicine. We grant that *writers* might be found for such an undertaking—but not *readers*. In English literature, as well as in Irish agriculture, there is an important class of personages, who may be denominated Middle-men—without whom the fields of science would lie waste. These personages have an especial eye to business—(and truly we do not blame them on this account)—by which vigilance they are enabled pretty clearly to foresee whether or not the Sub-letting process will flourish—in other words, whether or not a book will *sell* after the expensive preliminary of *printing*. When we connect *expense* with *printing*, and make no allusion to the expenditure of brains in composition, we are influenced by the prevailing ideas of the Middle-men, who assure us that there is little or no expenditure of brain on these occasions, in general—and that where there *is* such expenditure, the proprietor cannot possibly vest his intellectual property in better stock than that of —the Public Funds.

The author of the work before us appears to have been as fortunate in the choice of a subject of investigation, as industrious in the collection of materials for its construction. No one who looks abroad over the surface of this globe, even on maps or in books, and observes the infinite variety of form, colour, constitution, and moral character, which the different nations, tribes, and races present, can, for a moment, doubt that all, or almost all these varieties (we mean the *physical* peculiarities) have been worked by the slow hand of time, through the instrumentality of *climate*, in the extended sense of the word. If, again, we narrowly inspect the different localities of the same climate or country, we shall see corresponding physical peculiarities impressed on the forms, and on the temperaments of the inhabitants. This being the case, who can doubt that considerable influence may be exerted on the human constitution by the transition alone from one climate to another of a different character? The *injurious* effects of these transitions are, unfortunately, but too familiar to our eyes. Look at those who have returned from 21 years transportation among the jungles, swamps, and burning sands of the East—or the deadly mangrove-shores or savannahs of the West. They have dearly paid for their pagodas and sugar-hogsheads! They have exchanged the clear complexions and rosy tint with which they departed from the chalky cliffs, for—what shall we say?—the tough, the torpid, and the turmeric coat of the turtle! But have we no bright side to the picture? Is it not probable that the azure skies and balmy airs of Italy—the clear, elastic, mountain breeze of the Alps and Pyrennees—the purified zephyrs of the Azores, may, in many disordered states of the British constitution, contribute materially to the renovation of health and tranquillity of mind? We think there cannot be a doubt of this fact. Even when disorders of function have passed the awful boundary, and changed into diseases of structure, some mitigation of suffering, some retardation of the final event, some improvement in the condition of impaired organs, may possibly be effected by a judicious change of climate. But where are we to look for any precise information, any definite ideas as to the climates—and still more the localities of climates, adapted to particular diseases, or temperaments of constitution? The long and sanguinary warfare which *morally* insulated the inhabitants of this country, and banished them from the Continent, not only prevented the acquisition of all medico-topographical knowledge of this kind, but rendered it useless if it could have been obtained. The short period of peace has given an astonishing impulse to British medical travellers, and has accumulated more information on the subjects of medical topography and the effects of climate (as far as Continental Europe is concerned) than the preceding half century. Dr. Clark, like several of his naval and military brethren, has had ample opportunities of studying the effects of climate, in some of the fairest regions of the earth, and the present work will amply prove that he has not suffered those opportunities to pass unavailed.

"Et mores hominum multorum vidit et urbes."

Besides the South of Europe, Dr. Clark has recently made himself acquainted with the milder parts of England to which invalids resort, or might resort with advantage, as a succedaneum for the more brilliant but expensive and inconvenient skies of Italy or France.

The work is divided into two parts—in the first of which, the author has endeavoured to determine the general physical characters of the milder climates of southern Europe and England—to point out the manner in which the climate of different places is modified by local circumstances—and to compare these places relatively to their influence on disease. In the second part, Dr. C. has given some account of the principal diseases which are benefited by a mild climate. Although we think that the two parts would have been more beneficially blended in one, by which much repetition and reference from one part to another would have been avoided; yet we can offer no serious objection to the plan which Dr. C. has judged it prudent to adopt. The two great classes of disease into which our author has gone somewhat minutely, are pulmonary consumption and disorders of the digestive organs. The former class has always been associated in the mind with change of climate: but it is very questionable whether the digestive organs are not more improved by "a continuous and rapid change of climate, as well as of scene," than the pulmonary apparatus.

After some introductory remarks on the difficulty of investigating the physical influence of climate—and even the physical characters of the same, our author proceeds to a kind of natural grouping or classification of the different places to which invalids resort.

"Thus, we find that the South-West of England (with the exception of the extreme western point of the peninsula of Cornwall, which has a climate peculiar to itself) resembles very closely the South-West of France; that the South-East of France is opposed in every respect to the South-West of France; that the climate of Italy differs from both; that the climate of Nice partakes of the qualities of the Italian as well as of that of Provence; and that Madeira, or the climate of the Eastern Atlantic, can be classed with neither of the foregoing." 16.

### I. ENGLAND.

Before crossing the Alps or the Atlantic in search of a salutary clime, we ought to investigate the resources of our own. Dr. C. is inclined to think that "England possesses advantages which have not been made so fully available in this way, as they might have been:"—in short, that many invalids have gone abroad in search of that which was at their own doors. It is probably with health as with happiness—which—

——— Still so near us, yet beyond us lies,
O'erlooked, seen double by the fool and wise!

Or still more likely it is the journey which we undertake in quest of health, and the cheering hope of attaining it, rather than the spot of ground where it is said to reside, that produce such salutary effects on the invalid.

Dr. Clark starts his inquiries from the great Babylon—"the most important point in our survey"—that MART of every thing estimable and detestible—and, (as *physical character* is the subject of investigation) that MONOPOLY of carbon and hydrogen—whose AIR is chiefly composed of powdered flint and sublimed sea-coal—and whose waters, like the blood, are perpetually running in a circle—from the mouth to the anus—from the anus to the mouth!

The mean annual temperature of London is 50°, 39′, being about a degree and a half above that of the environs, with the exception perhaps of Brompton and Chelsea, which are confessedly sheltered. That London is, comparatively speaking, a healthy metropolis, we can have no doubt. A thousand circumstances conspire to its salubrity, when its forty or fifty millions of inhabitants are considered;* yet, as a point de depart, we must, pro formâ, set down the modern Babylon as possessing the minimum of salubrity.

### SOUTHERN COAST.

Various places along this extensive sea-board are frequented by invalids, in search of milder seasons than the interior of England presents. Of these the principal points are Hastings, Brighton, Chichester, Gosport, Southampton, and the Isle of Wight. The mean temperature of the southern coast during the Winter months, is only one or two degrees *above* that of London —in the Summer it is about the same ratio *below* the heat of the metropolis. The range of temperature, too, is less on the coast than in the interior—in short, the climate is more steady. The general character of the line of coast from Worthing to Southamptom is said, by Dr. Clark, to be "humid and heavy," without protection from winds, and, in many parts, *aguish.* After alluding to this aguish disposition, our author informs us that CHICHESTER "is certainly the best Winter residence for that class of invalids likely to be benefited by a climate of *this* kind." We confess our opinion that the doctors of this malarious district are much more likely to be benefited by the climate than any class of invalids who may resort thither.

---

* This estimation may startle those who only calculate on men, women, and children, as the inhabitants of a town or city. We are not inclined to maintain that a horse deteriorates the atmosphere as much as a man. On the contrary, we believe him to be a much more cleanly, and a much more useful animal. But if we contemplate the chain of animated beings in London, from the "*half*-reasoning elephant" down to the gnat, and up again to the *false*-reasoning, and, too often, *no*-reasoning lord of the creation, it will be acknowledged that, if we had put billions or trillions for millions, we should not have been far wrong.

Hastings, Brighton, and the Isle of Wight, are excepted from the character of climate above described, and are separately treated of by the author. Of Hastings we need say little, since Dr. Harwood's work has made the medical topography and climate of that watering place well known. We shall make room for a short extract.

"It must be admitted, indeed, that one of the principal disadvantages of Hastings, is its confined situation, by which its climate is limited to a small local extent, owing to the close manner in which it is hemmed in on the sea by the steep and high cliffs which rise immediately behind it. This circumstance, moreover, gives the climate of this place a peculiar character relatively to its effect upon certain constitutions. With respect to the merits of the climate of Hastings, it may be observed, that its superiority in winter appears to be confined chiefly to the months of January and February, but that during these and the spring months, it has the advantage of all the other places frequented on the South Coast in warmth and shelter from cold winds, (with the exception, perhaps, of Undercliff, in the Isle of Wight,) and, consequently, during this period affords the best residence for those invalids to whom the climate of this coast is adapted. During the autumn months, and even December, the climate of Brighton appears to be somewhat warmer and more steady than that of Hastings.

"In its effects on disease, the climate of Hastings corresponds with that of the South Coast generally: it is, however, according to my experience, unfavourable in nervous complaints, to persons subject to headache, and to languid or relaxed habits. For persons also who have suffered from ague, I should consider Hastings as a very doubtful residence. There is a degree of closeness and confinement in the atmosphere of the lower and more sheltered parts which is unfavourable to the former class of invalids; and agues are not unfrequent about Hastings." 27.

BRIGHTON.

The climate of this favourite spot is the reverse of Hastings, as far as closeness is concerned—being dry, elastic, and bracing. To people of nervous or relaxed habits, therefore, it is much more favourable than its more sheltered neighbour. The Autumn and beginning of Winter are supposed to be the best seasons for Brighton.

"The early part of the spring is the worst season at Brighton, its exposure to the north-easterly winds rendering it an unfavourable climate at this season, to all persons disposed to pulmonary or other diseases accompanied with much general or local irritation. As examples of such, I may mention affections of the trachea and bronchia of the dry irritable kind, and dyspepsia arising from an irritated state of the stomach.

"For the greater number of valetudinarians who frequent the Southern coast, it is probable that the autumn and beginning of winter may be passed with the greatest advantage at Brighton;—January and February at Hastings; and that some sheltered situation in the interior, is preferable to any part of the coast in the spring." 28.

ISLE OF WIGHT.

This "Garden of England," as it has been called, appears to be more resorted to for pleasure by the tourist, than for health by the invalid.

"The only part of this island well adapted as a winter residence for invalids, is that denominated *Undercliff*, which comprehends a tract of country about six miles in length, and from a quarter to half a mile in breadth, situated on the South-East coast, between the villages of Bonchurch and Niton. This singular district consists of a series of irregular terraces, formed by fragments of rocks of chalk and sandstone, and the alluvia of a rich loam, which have been detached from the cliffs and hills above, and deposited upon a substratum of blue marl. The whole of the Undercliff, which presents in many places scenery of the greatest beauty, is dry and free from moist or impure exhalations, and is completely sheltered from the north, north-east, north-west, and west winds, by a range of lofty downs, or hills, of chalk and sandstone, which rise boldly from the upper termination of these terraces, in elevations varying from four to six and seven hundred feet; leaving Undercliff open only in a direct line to the south-east, and obliquely to the east and south-west winds, which rarely blow here with great force. 'On this part of the coast,' says Dr. Lempriere, 'we have a climate as favorable to the invalid as any part of England can afford. This is proved not only by thermometrical observation, but also by the state of vegetation during the colder months of the year, when the myrtle, geranium, and many other exotic plants flourish luxuriantly in the open air; and that even in seasons when the severity of the frost has destroyed the green-house plants in the north side of the island, although placed in sheltered apartments. Snow is rarely seen, and frosts are only partially felt here."

"The mildness of the climate, the dry nature of the soil of Undercliff, and the extent to which it is sheltered from cold winds by affording ample space for exercise, are great advantages to invalids threatened with pulmonary disease, or others to whom exercise in the open air, in a mild climate, is desirable. In this respect, it is probably superior to any place in this line of coast. The principal objections to it, as a residence for invalids, are the scantiness of accommodations, and its distance from medical advice. On this account, it is perhaps, at present, rather calculated for the retreat of the valetudinarian, who does not stand in need of much medical attendance, than for the real invalid." 30.

### SOUTH WEST COAST OF DEVON.

The warmer parts of this district have a temperature nearly two degrees higher than the coasts of Sussex and Hampshire, and three or four above London. It is in the months of November, December, and January, that this difference is most remarkable, amounting, on an average, to five degrees above London. This difference, too, is chiefly observable in the night. Torquay is now becoming the favourite resort for invalids.

"The general character of the climate of this coast, is soft and humid. Torquay is certainly drier than the other places, and almost entirely free from fogs. This drier state of the atmosphere probably arises, in part, from the lime stone rocks, which are confined to the neighbourhood of this place, and partly from its position between the two streams, the Dart and the Teign, by which the rain is in some degree attracted. Torquay is also very remarkably protected from north-east winds, the great evil of our spring climate. It is likewise well sheltered from the north-west. This protection from winds, extends also over a very considerable tract of beautiful country, abounding in every variety of landscape; so that there is scarcely a wind that blows, from which the invalid will not be able to find a shelter for exercise either on foot or horseback. In this respect, Torquay is much superior to any other place we have noticed. It possesses all the advantages of the South-Western climate in the highest degree, and, with the exception of its exposure to the south-west gales, (one of the evils of this coast,) partakes less of the disadvantages of it than any other place having accommodations for invalids. Of the places on this coast frequented by invalids, Dawlish perhaps deserves the preference after Torquay;—it is less dry and more exposed to Easterly winds than the latter place. Exmouth is tolerably well sheltered, but damp and subject to fogs. Sidmouth, as will be perceived by a reference to the tables, possesses very little of the characteristic qualities of this climate. It is open to the valley of the Sid, and exposed to currents of cold wind from the mountains whence this stream takes its rise. It is therefore inferior to all the other places as a winter or spring residence. It may however be an agreeable summer or autumn watering place." 33.

The interior of Devonshire suffers considerably less from violent winds and storms than the coasts; and some of its sheltered vallies may probably afford protection from the East winds during Spring—but, in Winter, its temperature is considerably lower than the coast.

"The climate of the coast of Devonshire is found very beneficial in various forms of disease. I have known it serviceable in chronic affections of the throat, trachea and bronchia, proceeding from irritation, or a low degree of inflammation of these parts, and attended with a dry cough, or with little expectoration; likewise, in an irritable or morbidly sensitive state of the stomach, and in hypochondriacal affections, the consequence of such a state. In dysmenorrhœa and all nervous sympathetic affections dependant on that disorder; in a highly sensitive state of the nervous system, and in most diseases of general irritation, advantage may be expected from this climate. On the other hand, it certainly exerts an unfavourable influence on nervous headachs, and on all nervous complaints arising from relaxation or want of tone of the nervous system; it is injurious also in *pure dyspepsia, when the tone and sensibility of the stomach are below par, as indicated by pale lips, a pale clammy state of the tongue, and languid circulation;* and it will be found no less unfavourable in menorrhagia, in leucorrhœa, and all diseases accompanied with much general relaxation of the system, or with much discharge from the affected organs."* 35.

In respect to CONSUMPTION, our author here, as almost every where else, has maintained a rigid caution in pronouncing any thing positive.

"But this much I may venture to advance, that as the invalid will be exposed to less rigorous cold, and for a shorter season,—will have more hours of fine weather, and, consequently, more exercise in the open air,—he gives himself a better chance by passing the winter here, than he could have in any more northern part of our Island. To compare it, also, in this respect, with the climate of the southern continent of Europe, is no easy task. In the South, the invalid has finer days, a drier air, and more constant weather; but the transitions of temperature, though less frequent, are more considerable. In the nights I believe invalids are often exposed to severer cold than here; and this arises partly from the great range of temperature, and partly from the imperfect manner they are protected from the cold of night, by the bad arrangement of the houses, chimnies, &c." 35.

PENZANCE next engages our author's attention—and Dr. Forbes's publi-

---

* We have marked a passage in Italics, in the above quotation, because we believe the worthy author has involved himself in a degree of inconsistency—as well as of error, by certain expressions there. In the foregoing passage we are told that, in *pure* dyspepsia, the *sensibility* of the stomach is "below par." Now, on turning to page 181 of the work, we find the morbid conditions of the stomach in dyspepsia divided into two kinds:—"1mo. An irritated state of the mucous surface of the stomach of an inflammatory character; and, 2do. A *highly increased degree of sensibility of the nerves of the same part,* accompanied mostly with a loss of tone of the whole viscus. The latter affection constitutes the *pure* or nervous dyspepsia." The inconsistency in these two passages we did not expect in so cautious a pathologist as Dr. Clark. But we shall have an opportunity, in the second article of this analysis, to enter more fully on the subject of dyspepsia—a subject which occupies a considerable part of the volume under review.

cation on the climate of that peculiar spot, furnishes, of course, the materials of this section. It is only necessary to remark here, that—

"On examining the progression of temperature for the twenty-four hours, at these two places, we find that, in winter, it is during the night that the greater part of this difference of temperature occurs; Penzance being nearly on an average, six degrees and a half warmer than London during the night; and only little more than three degrees warmer during the day. This distinction ought to afford matter for the physician's consideration. But this equal distribution of heat throughout the year at Penzance, which we have compared so advantageously with that of London, is still more striking when compared with that of the South of Europe. Madeira is the only climate which we have examined that is superior to Penzance in this quality." 39.

It appears that the whole advantage of Penzance, as compared with the South of Europe, occurs *during the night*, in Winter. It is during the day only, that the South of Europe claims superiority as to temperature. Thus, in Winter, at seven o'clock in the morning, there is little difference between Rome and Penzance; but, at two o'clock in the afternoon, the temperature of the Italian metropolis is seven degrees above that of the Land's End of England.

"In the duration of the same temperature, as shown by the mean variation of successive days, the climate of Penzance excels all the northern climates, and nearly equals Rome and Nice in this respect; but as compared with Madeira, its temperature from day to day varies twice as much." 41.

In Spring, Penzance loses its superiority of climate. In April and May, it appears inferior to the South-western coast—and very much so to the South-west of France. In Penzance there falls about twice as much rain as in London. The storms, too, are violent and frequent—which frequency and violence, says Dr. Clark, "constitute one of the greatest disadvantages of its climate." The following quotation from the recent edition of Laennec, by Dr. Forbes, will shew the present opinion of Dr. F. on the climate of Penzance, as far as pulmonary phthisis is concerned.

"During a residence of five years," says Dr. Forbes, "at Penzance, in Cornwall, a place much frequented by consumptive patients on account of the mildness and equability of its temperature, I had extensive opportunities of observing the effect of changs of climate in phthisis; and I am sorry to say that, in the greater number of cases, the change was not beneficial. This result, however, must not, in fairness, be considered as derogating, in any considerable degree, either from the propriety of the practice, or the fitness of the situation; since it must be confessed that few of the invalids came to Penzance in that period of the disease when a cure could be expected, if indeed it were even possible. In no case of well marked tubercular phthisis did I witness a cure, or even a temporary alleviation, that could fairly be attributed to change of climate. In a good many cases, however, of chronic bronchitis, simulating phthisis, the health was greatly improved, and in some it was completely restored, from a state of great debility and seeming danger. In a few cases, also, of young persons who accompanied their diseased relatives, and in whom the hereditary predisposition was strongly marked, if there was not already evidence of nascent tubercles,—a great and striking improvement in the general health and strength followed within a short period after their arrival, and seemed fairly attributable to the combined influence of change of air, scene and habits."* 48.

---

"* Dr. Forbes' Translation of Laennec's Treatise on Diseases of the Chest. Note by translator, 3d Edit. p. 73."

Our golden dreams of Penzance then, are at an end! But we shall add Dr. Clark's own opinion.

"The consumptive cases in which the soft humid atmosphere of this place is likely to prove beneficial, are those in which the disease is accompanied with an irritated state of the mucous membrane of the lungs, producing a dry cough, or one with little expectoration.

"In idiopathic tracheal and bronchial diseases of the same character, whether complicated with asthma, or otherwise, and also in certain pure cases of the latter disease, it is likely to be very beneficial. When, on the contrary, there exists a relaxed state of the general system, or a disposition to copious secretion from the bronchial membrane, whether idiopathic or symptomatic of a tuberculous state of the lungs, or where hæmoptysis has occurred, I believe the climate of the Land's-end will generally prove injurious." 49.

WEST OF ENGLAND.

Of this line of coast, we shall only notice CLIFTON, because we have reason to know that it is a very salutary spot, and, from its topography, capable of affording considerable variety of shelter or exposure, according to the nature of the case. Dr. Clark does not appear to have seen Clifton himself, at the time his book was in the press, but we understood from him that a subsequent visit impressed him strongly with the salubrity of the place. Drs. Nott and Chisholm afford the information contained in Dr. Clark's work. Dr. N. observes—

"To no spot in our kingdom can the valetudinarian fly, or can the physician consign his patient, for a re-establishment of health impaired by disease, where the purpose is more attainable, than in the neighbourhood of St. Vincent's Rock. The upland habitations on Clifton Hill during the summer are delightful; and the sheltered situation of those round the Hotwells below, afford a warm residence in winter, equal at least to the boasted Coast of Devonshire; indeed it has one advantage over it, being free from its damp airs and mizzling rains." "Had Dr. Nott," observes Dr. Chisholm, "adverted to the influence of subincumbent lime stone, as constituting the salubrity of the atmosphere, his picture would have been more complete. The absence of every thing like marsh must certainly contribute greatly to the purity and wholesomeness of the Clifton air." "In the list of diseases admitted during four years into the dispensary, only one case of intermittent fever appeared, and that one was from the fenny district near Conglesbury, about twelve miles to the westward of Clifton." "The whole parish of Clifton," Dr. Chisholm further adds, "is a beautiful and romantic assemblage of woods, rock, water, pasture and down. It seems indeed singularly well adapted to the maintenance of health; the soil resting on immense beds of lime-stone rock, exposed to the southerly and westerly winds, for nearly three fourths of the year; with an atmosphere elastic, vivifying—not humid." 55.

Before taking leave of England and its climates, the invalid may naturally ask the profession what advantages are held out to him by the localities adverted to above? It may be observed that all those places are warmer in Winter than England generally—much warmer than the colder parts of it. Thus there is nearly as much difference between the South of Scotland and South of England, as between England and the South of Europe. An inhabitant of one of the coldest parts of our island therefore, would feel the influence of the climate of Hastings or Torquay, as much as a Londoner would feel the influence of the climate of Nice. As far as regards the influence of these milder spots of England on the scourge of our country—

phthisis, the author refers to the article on consumption in the second part of the work, and which we cannot here notice. The following extract is couched in Dr. Clark's characteristically cautious manner.

"I may remark here, that it is (with a few exceptions only) as a means of preventing the occurrence of tubercular disease in the lungs, when threatened, or of checking its progress in its early stages, that much benefit can be expected from any climate. With this view, the preference to be given to any of the milder situations in England, in such cases, must depend greatly on the nature of the person's constitution, and partly also on the other morbid states with which the consumptive tendency may be complicated. Keeping these circumstances (the whole of which have been noticed in the articles referred to) in mind, the situation which admits of the invalid's being most in the open air without risk from cold, should in general be preferred. The selection will, I believe, lie among the following places, as winter and spring residences,—Torquay, Undercliff, Hastings and Clifton; and perhaps, in the generality of cases, they will deserve the preference in the order stated. But I wish to be understood as speaking with much diffidence on this subject." 59.

### FRANCE.

There is no more common feature or propensity in the human mind than to undervalue that which is familiar to us, or easily obtained; and to over-estimate that which is foreign, or with difficulty procured. A man will come from Leeds or Manchester to London for medical advice, and prefer five or ten minutes routine questions, with a milk and water prescription, to a careful and accurate investigation of his complaint in his native place of residence with appropriate remedies. And strange to say, he will often derive more benefit from the very same medicine, if prescribed in London, than if directed in Yorkshire. The influence of the imagination and of the journey can alone explain this enigma. So it is with climate. The hope of benefit from a foreign sky—the exercise of travelling to the new climate—and the impression of moral emotions from new scenes and new faces, often do much more towards the restoration of health than all the barometrical, thermometrical, or hygrometrical peculiarities of the selected residence.

The South of France has long been supposed to be the grand restorative of English constitutions, and many a sufferer has migrated thither to consign his or her mortal remains to unconsecrated earth. More correct views have lately been taken of the effects of climate, and of the diseases for which change of climate is desirable. The South of France may be classed under two divisions of climate, essentially differing from each other—the South-eastern, and the South-western.

### WEST AND SOUTH-WEST.

This extends from Britanny to Bayonne, including L'Orient, Nantes, Bourdeaux, Pau, and Toulouse. The islands of Guernsey and Jersey are in the same range—or they may be considered as intermediate between that and the South-west coast of England. The S. W. coast of France, though less warm than the S. E. is yet more equal in its temperature, the range of which is less extensive throughout the year, and throughout the 24 hours. But it is more changeable from day to day. The mean annual temperature of the S. W. coast is about 55°—or 6° above that of England generally—

4° higher than the S. W. of England—3° below the S. E. of France—4° below Italy.

"The days are not so fine as in the south-eastern parts of the kingdom, but the nights are not so cold in relation to the days. The climate may be characterized as soft, relaxing, and rather wet. Hence it is suitable for complaints to which the south-east of France is injurious, particularly gastritic dyspepsia, (or dyspepsia depending on an inflammatory state of the stomach,) and the dry bronchial irritations. In that class of consumptive patients, therefore, in whom the disease is complicated with either or both of these affections, and in whom, consequently, there is a great susceptibility to the influence of dry, keen winds, this climate will generally agree. Laennec found the southern coast of Brittany very favourable to consumptive patients; and he also observed that the proportion of consumptive diseases in this part of France, was comparatively small." 65.

PAU.—This romantic spot, the capital of the lower Pyrenees, is the only place which our author considers it necessary to notice in this range of climate. It is finely situated on a ridge of gravelly hills overlooking an extensive valley to the North. The Pyrenees rise gradually behind it, their higher range being nearly 40 miles distant. PAU is about 150 miles from Bourdeaux, and 50 from Bayonne. Dr. Playfair, now of Florence, resided at PAU several years, and favoured our author with an account of its climate, from which we shall make some extracts.

The mean annual temperature of this place is $4\frac{1}{2}$° above London—3° higher than Penzance—5° lower than Marseilles, Nice, or Rome—10° lower than Madeira. The range of temperature between the warmest and coldest months is 32°—which is 6° wider than that of London or Rome.

According to Dr. Playfair, the good qualities of PAU may be summed up as follows:—

"Calmness, moderate cold, bright sunshine of considerable power even in winter, a dry state of atmosphere and of the soil, and rains of short duration. Against these must be placed—changeableness, the fine weather being as short-lived as the bad; rapid variations of temperature, within moderate limits however; and heavy rains in autumn and spring.

"Pau is upon the whole healthy. Measles, scarlatina, and hooping cough, are generally mild, and croup almost unknown. Intermitting and bilious fevers, and rheumatism, are the most prevalent diseases. Rheumatism, according to a native author, is the only disease that is very common; it exists almost as an endemic, and stimulates or complicates almost all the other diseases. Goitre is also very common among the peasantry. The intermitting fevers occur chiefly among those of the peasants who frequent the low damp grounds in the neighbourhood. Scrofula is rare, and consumption not a common disease.

"There are several circumstances in the climate of Pau which render it a favourable residence for a certain class of invalids. The atmosphere, when it does not rain, is dry, and the weather fine, and there are neither fogs nor cold piercing winds. The characteristic quality of the climate, however, is the comparative mildness of its spring, and exemption from cold cutting winds. While the *winter* is 3° colder than the warmest parts of England, and 6° colder than Rome, the *spring* is $5\frac{1}{2}$° warmer than the former, and only $2\frac{1}{2}$° colder than the latter. The mildness of the spring, and its little liability to winds, render this place favourable to chronic affections of the larynx, trachea and bronchia. In gastritic dyspepsia also, Dr. Playfair has found it beneficial, and he has seen it useful in a few cases of asthma.

"Upon the whole, Pau appears to be one of the most desirable winter residences in the south-west part of France, for invalids labouring under chronic affections of the mucous membranes. In the same class of diseases, the mineral waters of the Pyrenees are also very beneficial; and it may be convenient, and adviseable, for the invalid who has derived benefit from a course of these waters, to pass the winter at Pau, with a view of returning to them in the following season. These waters may also be easily transported to Pau, and used occasionally during the winter. Those of *Bonnes*, which retain their qualities well, and are among the most efficacious of the waters of the Pyrenees, are at no great distance.

"With delicate children, Dr. Playfair found the climate agree well, especially when they removed to the mountains during the summer.

"Invalids labouring under, or liable to attacks of rheumatism, should, of course, avoid Pau. In bronchial diseases, also, when accompanied with much general relaxation of the system, and with copious expectoration and dyspnœa, the climate will not in general prove beneficial; and Dr. Playfair considers it too changeable in consumptive diseases.

"Invalids who mean to pass the winter at Pau should arrive there in the end of September, or very early in October. In selecting apartments, (which are not very numerous,) they should recollect that it is of importance that these should have a southern aspect.

"In fixing the period for leaving Pau, the destination of the person must be taken into account. If the object is to return to England, he may leave it in May; if he means to spend the summer among the Pyrenees, he should not leave it before June. The best season for using the mineral waters of the Pyrenees commences about the first of July." 73.

### SOUTH-EAST OF FRANCE.

Various places in this quarter have been, at different times, extolled as affording a good Winter climate for *consumptive patients*, though recent experience has proved that this, like many other medical opinions, has been not merely erroneous, but injurious.

"That the country which has always been infested by the terrible *Circius*, should have been chosen for the residence of the delicate and sensitive sufferer from pulmonary disease, is a striking proof of the very loose observations upon which medical opinions respecting climate have been formed. How this practice of sending consumptive invalids to the south-east of France, originated, it is not of importance to inquire; that it is founded on error, I think, I shall be able to prove, by a reference to the physical characters of the climate, the actual prevalence of consumption among the inhabitants, and, I may add, the total want of success which has attended the measure.

"The mean annual temperature of *Provence* generally, is 58°; that is, about 7° warmer than the south-west of England, 3° warmer than the south-west of France, and about a degree below Italy, including the climate of the lower Apennines. Its *winter* temperature is 43°; being only 1½° above the south-west of England, and 1° above the south-west of France, while it is 3° under Italy. The *spring* temperature is 55°; namely, 6° above the south-west of England, 1° above the south-west of France, and 2° below Italy. The temperature is distributed very unequally through the year; the difference of the mean of the warmest and the coldest months being 35°; this in the south-west of England is 22°, in the south-west of France 30°, in Italy 32°, and in Madeira only 14°." 74.

Dryness is one of the most remarkable characters of the climate of Provence. There is not half the annual fall of rain there that there is in Penzance. The annual number of days on which rain falls in Provence is 67, while in London it is 178.

"Again, in Provence (at Toulon) the quantity of water evaporated annually, is 40 inches, while at Paris it is 32 inches, at Gosport 25, and at London only 24. When these circumstances are taken into consideration, together with the high mean temperature of the place, Provence must appear the driest district of Europe. Indeed, the dry nature of the soil, and the bare parched aspect of the country, bespeak such a climate.

"The general character of the climate of the South-East of France, therefore, is dry, hot, harsh, and irritating. Absolutely warmer than our own Island, and the south-western parts of France, its temperature is distributed through the year and through the day with great irregularity. It has a much wider range of temperature than our own climate; this being, when compared to that of England, as three to one for the year, and as two to one for the day. Sometimes the winter is very rigorous. In 1709, the ports of Marseilles and Toulon were frozen over; and, indeed, in ordinary years, the orange-trees are occasionally killed by the cold in the most sheltered parts of Provence. The temperature no doubt remains more steady from day to day than our own; but its changes, though less frequent, are more sudden and extensive.

"This tract of country is subject also to keen, cold northerly winds, especially the *mistral*, which prevails during the winter and spring, and is most injurious to pulmonary diseases.

"Although decidedly improper for consumptive patients, and for those labouring under irritation of the mucous membranes of the digestive or pulmonary organs, more especially irritation of the stomach, larynx, or trachea, this climate may prove useful to invalids of a different class. On persons of a torpid, or relaxed habit of body, and of a gloomy, desponding cast of mind, with whom a moist relaxing atmosphere disagrees, the keen, bracing, dry air of Provence, and its brilliant skies, will often produce a beneficial effect. In some cases of chronic intermittent fevers also, it proves very favourable." 76.

The distinctive characters of Provence generally prevail more or less in the different places resorted to by invalids. The following is the order in which, Dr. Clark thinks, these places ought to be preferred; viz:—Hyeres, Toulon, Marseilles, Montpelier, Aix, Nismes, Avignon.

It appears that in the Hôtel-Dieu of Montpelier, in the year 1763, the total number of patients was 2756, of whom 154 died. Of these last, 55 were phthisical patients—in other words, more than one third of the deaths was from consumption! It is unnecessary to adduce further evidence of the unfitness of Montpelier as a refuge for the phthisical invalid.

Marseilles is but little entitled, according to Dr. Clark, to claim any exemption from the general character of the climate of Provence. It is open to the full influence of the cold winds of the country—and especially to the mistral. There is no place in the neighbourhood of the city where invalids can take exercise. Aix is still worse.

Hyeres. This little town is agreeably situated on the southern declivity of a hill, about two miles from the mediterranean shore, and twelve miles from Toulon. It is the least exceptionable residence for the pulmonary invalid in Provence. It is in some degree protected from the northerly winds, and is situated in a beautiful open country, with orange trees under the town. There are some spots sheltered from the mistral, where the invalid might enjoy several hours in the open air almost every day; but the difficulty is to reach these at the proper time. The invalid dare not go out without a close carriage, and the roads will not admit of such a vehicle.

### Nice.

The climate of this celebrated spot approximates more to that of Provence than to that of any other. Its mean annual temperature is 59°, being 9° warmer than London—7° above Penzance—1° below Rome—and 5° colder than Madeira. The mean temperature of Winter is 48°—that of Spring 56.° The temperature throughout the year is more equally distributed at Nice than at any place in the South of Europe, except Rome and Cadiz—the difference between the warmest and coldest months being only 28 degrees—and the mean difference of successive months only about 4 degrees. The range of temperature for the day is also less at Nice than in any part of the South of Europe—and, in steadiness of temperature it ranks next to Madeira.

"The weather at Nice during the winter is comparatively settled and fine, the atmosphere being generally clear, and the sky remarkable for its brilliancy. The temperature seldom sinks to the freezing point, and when it does, it is only during the night; so that vegetation is never altogether suspended. Indeed, at Nice, winter is a season of flowers, the dryness of the air rendering the same degree of cold less injurious to them, than it would be in a more humid atmosphere. The mild and equable character of the climate of Nice depends in a great measure on its position, with respect to the neighbouring mountains and the sea. The maritime Alps rise immediately behind it, and form a lofty barrier which shelters it from the northerly winds during winter; while, on the other hand, the heat during summer is moderated by the cool sea-breeze, which prevails here every day, with a regularity almost equal to that of a tropical climate. 'Cet alizé mediterraneen,' says M. Risso, 'toujours doux, frais et tranquille, s'eleve periodiquement vers neuf à dix heures du matin, cesse souvent vers les quatre heures après midi, et s'étend dans l'interieur de nos Alpes rarement au delà de huit myriamètres.' These circumstances explain the small annual range of temperature at this place, already noticed, and which a reference to the table in the appendix will show to be much less than at most parts of Italy.

"Notwithstanding the extent, however, to which Nice and its environs are encircled by mountains, (and it is so in a great measure from W. S. W., to E. S. E.,) it is by no means exempt from cold winds during the winter, and still less so during the spring. The easterly winds are the most prevalent during the latter season. They range from east to north-east, frequently blow with considerable force, and are often accompanied with a hazy, cloudy state of atmosphere. Sometimes this wind sets in towards the forenoon, at other times not until the afternoon. When the early part of the day is fine, it never should be lost for exercise; as the afternoon frequently proves cold and windy, after a calm, mild, morning.

"From the north-west, or mistral, which is the scourge of Provence, Nice is pretty well sheltered. The force of this wind seems to be broken, and directed to the southward by the Estrelles, a chain of mountains between Frejus and Cannes. But, although the mistral is never experienced in its full power at Nice, or only towards its termination, when it takes a more westerly direction, (*la queue de la Mistral*, as it is called,) the keen, dry quality of the air is very sensibly felt whilst it prevails. It sets in generally about two or three o'clock in the afternoon, and is not of long duration. It seldom blows strong directly from the north, though the air is very sharp when the wind is in that quarter. The northerly gales appear to pass obliquely over Nice. The sirocco rarely blows, and when it does, it is gentle, and not unpleasant to the feelings of invalids in general. But the sharp, chilling, easterly winds are the greatest enemy the invalid has to contend with at this place; and the prevalence of these during the months of March and April is admitted, I believe, by all who have felt them, to form a great objection to this climate, especially in pulmonary diseases.

"The climate of Nice is altogether a very dry one. Rain falls chiefly during particular seasons. From the middle of October to the middle of November it generally rains a good deal; also about the winter solstice there is commonly some rain, and again, after the vernal equinox. The quantity of rain that falls during the year has not been accurately estimated.

"Upon the whole, in the physical qualities of its climate, Nice possesses some advantages over the neighbouring countries of Provence and Italy, inasmuch as it may be said to be free from the sirocco of the latter, and protected from the mistral of the former." 87.

Nice is a healthy place, being exempt from endemic diseases, and rarely troubled with epidemics. Catarrhal affections and inflammation of the lungs rank among the most prevailing diseases. The latter is very common in the Spring, and is generally complicated with irritation of the digestive organs. The proportion of deaths from pulmonary consumption in the Hospital of Nice, is said to be about one seventh of the whole mortality. "Gastric irritation appears to be the prevailing endemic disorder of the place." To Dr. Skirving, who has resided many years at Nice, our author is much indebted for the results of his observation on the influence of the Nicean climate on disease.

"In *Consumption*, the disease with which the climate of Nice has been chiefly associated in the minds of medical men in this country, little benefit I fear is to be expected. When this disease is complicated with an inflammatory, or highly irritable state of the mucous membranes of the larynx, trachea, or bronchia, or of the stomach, Nice is decidedly an unfavourable climate; and, without extreme care on the part of such patients, and a very strict regimen, the complaint will in all probability be aggravated by a residence here. Indeed, the cases of consumption which ought to be sent to Nice are of rare occurrence. If there are any such, it is when the disease exists in torpid habits, of little susceptibility, or not much disposed to irritation; and when it is free from the complications which have been just mentioned. Even the propriety of selecting Nice as a residence for persons merely threatened with consumption, will depend much upon the constitution of the individual. Dr. Skirving has met with cases which leave no doubt on his mind, that a residence for one or two winters often proves of advantage, as a preventive measure, in young persons threatened with this disease; and even in some cases when there was every reason to believe that tubercles already existed in the lungs, the climate has appeared to be useful. But in the advanced stage of consumption, his opinion, founded on eight years' experience, accords with what has been already stated; and this is still further supported by the testimony of Professor Foderé of Strasbourg, who resided six years at Nice. Indeed, sending consumptive patients in this stage to Nice, will in a great majority of cases prove more injurious than beneficial.

"In *Chronic Bronchial* diseases, which often simulate phthisis, very salutary effects are produced by a residence at this place. Such patients generally pass the winter with little comparative suffering from their complaint, and with benefit to their general health. They are here able to be much in the open air, whereas if they had remained in England, they would in all probability have been confined during the greater part of the winter to the house. The particular kind of bronchial disease most benefited by a residence at Nice, is that accompanied with copious expectoration, whether complicated with asthma *(humoral asthma)* or otherwise; and in the chronic catarrh of aged people it is particularly beneficial. This variety of bronchial disease is directly the reverse of that which is benefited by the south-west of France and of England: and I think it important here to remark, that unless the distinctions which I have pointed out in bronchial diseases, and their complications, are attended to, great errors must be committed in selecting a residence for such patients. But for fuller information on this subject, I must refer the reader to the article on 'Bronchial Diseases.'" 91.

Dr. C. observes that, in what is commonly called dyspeptic complaints, and the numerous train of hypochondriacal and nervous symptoms which often originate in such a source, Nice is beneficial.

"But here, again, it is necessary to distinguish the particular character of the affection. The cases of dyspepsia most benefited, are those accompanied with a torpid, relaxed state of the system, with little epigastric tenderness, or any of those symptoms which denote an inflamed or very irritable state of the mucous membrane of the stomach. Where the latter state is the cause of the dyspeptic symptoms, Nice will decidedly disagree; indeed, as I have already observed, a degree of this affection is almost endemic there." 92.

In all cases where there is great relaxation and torpor of the constitution, the climate of Nice is extremely useful. Also in a numerous class of patients, whose constitutions have been injured by a long residence in tropical countries, by mercury, &c. and in which a dry and rather exciting climate is indicated, Nice will prove favourable.

"In stating its general influence on the animal œconomy, I would say—that the climate of Nice is warm, exhilirating, and exciting, but upon the whole, irritating,—at least to highly sensitive constitutions. It is extremely favourable to the productions of the vegetable kingdom, some of which flourish here in a degree of luxuriance that is scarcely to be equalled in the other parts of the south of Europe." 93.

Invalids at Nice scarcely ever reside in town. Some good lodgings, over-looking the terrace, are to be had; but the largest and best houses are situated in the suburb called the Croix de Marbre, and along the sea-beach, from the town to the ridge of the mountains bounding the plain on the west. Invalids should arrive at Nice about the middle of October, or sooner—and should not leave it before the beginning of May.

### ITALY.

The poetical representation which Virgil drew of the climate, soil, and skies of Italy, has, one would suppose, left a prepossession on every classical mind in favour of that interesting region of the earth. Much do we fear that the climate of Italy, though beautiful to the eye and genial to the senses, is not the paradise which it is thought to be.

The observations of Dr. Clark are limited to that tract which is situated between the northern shores of the Mediterranean, and the southern base of the Apennines. The climate prevailing over all this region exhibits a great similarity of character, and differs, in several respects, from any of the climates already noticed. It is much warmer and less humid—"but subject to a greater range of temperature than that of the south-west of France." It is softer, less dry, and less harsh and irritating than that of Provence—suffering more from the heavy oppressive winds of the south—and less from the dry searching winds of the north.

The modifications of climate in Italy appear to result chiefly from the relative position of places in respect to sea and mountain. Genoa and Naples are in the vicinity of both, as the mountains there approach close to the Mediterranean. Pisa is only a few miles distant from Genoa, and close to the Tuscan hills. Florence is quite inland, and so embosomed in the Apennines as to have the character of its climate materially affected.

GENOA is hemmed in between a range of steep mountains and the sea, with little or no surrounding country adapted for exercise. It is therefore an unsuitable residence for invalids generally—there being but little in the character of the climate to recommend it. The Summer is hotter, and the Winter is colder than at NICE.

FLORENCE.—This is one of the most agreeable residences in Italy—but it is far from being favourable for an invalid—especially for a pulmonary invalid.

" Its situation among the lower Apennines, by which it is almost encircled, and the summits of which are covered with snow during the winter, together with its full exposure to the current of the valley of the Arno, renders Florence subject to sudden transitions of temperature, and to cold piercing winds during the winter and spring. Fogs, too, are more common here than at most parts of southern Italy. The winter temperature is upon the whole low, while that of the summer is high. The mean annual temperature is only 1½° below that of Rome; but this is owing to the great heat of summer at Florence; for the winter is only 4° warmer than that of London, and is nearly of the same temperature as the winter at Penzance. The difference between the mean temperature of the warmest and coldest months is 36°, which is one degree more than that of Provence. Nevertheless, although the daily, monthly, and annual range of temperature are very great, the climate is not more variable or unsteady from day to day than that of Rome, and is less so than that of Naples. The annual range of atmospheric pressure is greater than that of the neighbouring places. The annual fall of rain at Florence is 31.6 inches, but the number of days on which rain falls is only 103, being fewer than at Rome. In the winter the air is rather chilly, and loaded with moisture.

" I do not know any class of invalids for whom Florence offers an adviseable residence. My own opinion, founded partly on observation, and partly on the reports of invalids, perfectly accords with that of Dr. Seymour of London, and Dr. Down of Southampton, whose more extensive opportunities of observation during a long residence and extensive practice at Florence, make their testimony of greater value. 'The winter,' says Dr. Down, ' is extremely severe and wet, and the spring changeable, consequently highly injurious in complaints of the chest. The inhabitants are very subject to diseases of the lungs; and the acute inflammation of this organ, known under the popular name of *Mal di petto*, carries off yearly in the winter and spring an amazing number of them, particularly of the poorer classes, whose houses are ill calculated to afford protection against the cold and rains of these seasons.'* Florence is one of the places in Italy which agrees least with children. Intestinal worms are particularly common there, and dysentery is prevalent in autumn. Pellagra, a disease almost peculiar to Lombardy, is endemic in some parts of the neighbouring vallies of the Apennines.

" Florence itself is not subject to any endemic disease; and to persons not likely to suffer from the vicissitudes of temperature, which have been noticed, and who can support the great heat of summer, it holds out many inducements as a residence during the whole year." 102.

PISA has long had the reputation of being one of the mildest and most favourable climates in Italy for consumptive patients. It is still much frequented by invalids from this country. The town is built on the banks of the ARNO, about five miles from the sea. The surrounding country is flat, except towards the North, where it is somewhat sheltered by a range of hills, as also on the East by the lower Tuscan hills.

" Pisa is not so warm as Rome in winter, and is hotter in summer. In *winter* it is 7° warmer than London, and 2° warmer than Penzance. In *spring* it is 8° warmer than London, and about 7° warmer than Penzance. The range of temperature between day and night is very considerable. According to Professor

* " Observations on the Nature and Treatment of Fevers and Bowel Complaints, &c. in Greece, by J. Somers Down, M. D."

Piazzini, the fall of rain annually is very great, being 45.66 inches, which is nearly as much as falls in Cornwall. The climate of Pisa is genial, but rather heavy and damp. It is softer than that of Nice, but not so warm; less soft, but less heavy and depressing than that of Rome. For invalids who are almost confined to the house, or whose power of taking exercise is much limited, Pisa offers advantages over either Rome or Nice: The Lung' Arno affords a warm site for their residence, as well as a sheltered terrace for their walks. But they must be careful to confine themselves to it. They should not venture into the cross streets before April." 103.

Naples.—This beautiful city resembles Nice more than any other place, as far as its character of climate is concerned.

The Autumn and Winter are generally mild; but the Spring is subject to cold, sharp, irritating winds, rendered more hurtful to invalids by the heat of a powerful sun. The climate of Naples is much more changeable than that of Nice—and, if somewhat softer in the Winter, is more damp and wet. The sirocco is severely felt at Naples, and but slightly at Nice.

"The mean annual temperature is higher than that of Rome, Pisa, or Nice; but the annual range of mean temperature is very considerable—being 30°; whilst that of Nice is but 28°; and that of Rome only 26°. The distribution of temperature in the different months is more unequal than at Nice or Rome. The daily range of temperature is also very great, being 2° more than at Rome. The temperature likewise varies very much from day to day, as will appear from the following statement:—The mean variation of successive days at Naples is 3°.36; at Rome it is 2°.80; at Leghorn 2°.44; at Nice 2°.33. The annual range of atmospheric pressure is very small,—somewhat less than at Rome, and very considerably less than in the south-east of France. Rain falls less frequently at Naples than at Rome." 105.

Catarrhal affections are the most common popular diseases at Naples. Consumption is not very frequent, nor rapid in its course. Autumn is the most fatal season for the consumptive in this place. Rheumatism is very frequent, as are also cutaneous complaints and ophthalmia.

"Of Naples as a residence for invalids it is unnecessary to say much. Consumptive patients should certainly not be sent there. The circumstances which have been pointed out in its climate, sufficiently mark it as a very unsuitable residence for this class of persons; and to the list of its defects must be added that of its topographical position, which affords no proper places for exercise, without such exposure as would prove highly injurious to delicate invalids. For chronic rheumatism it is, as compared with Nice and Rome, certainly inferior. Naples is however well suited as a winter residence for those who are labouring under general debility and derangement of the constitution without any marked local disease. The beauty of its situation, the brilliancy of its skies, and the interest excited by the surrounding scenery, render it a very desirable and very delightful winter residence, for those who rather require mental amusement and recreation for the restoration of their general health, than medical treatment for any particular disease." 107.

Rome.—The climate of the everlasting city is mild and soft, but rather relaxing and oppressive. Its mean annual temperature is ten degrees higher than that of London, eight higher than that of Penzance, &c. In range of temperature Rome has the advantage of Naples, Pisa, and Provence—but not of Nice. Its diurnal range is nearly double that of London, Gosport, Penzance, and Madeira. In steadiness of temperature, from day to day, (in which our own country, with the exception of Penzance, is so deficient,) Rome follows Madeira, Nice, Pisa, Leghorn—but precedes Naples and Pau. With regard to humidity, Rome, though a soft, cannot be considered a damp climate. Compared with Provence and Nice, we find that one-third more rain falls at Rome, and on a greater number of days. It is, however, considerably drier than Pisa—and very much drier than the south-west of France.

"At Penzance there falls about one-third more rain than at Rome, and the number of rainy days is also about one-third greater. This circumstance, together with the greater evaporation going on at Rome, owing to its higher temperature, must make a considerable difference in the hygrometrical state of the atmosphere, at the two places. Rome is not so dry as Madeira; as there falls one-sixth more rain at the former place, and the proportion of wet days is as 117 to 73. From these comparisons, it would appear that the climate of Rome, in regard to its physical qualities, is altogether the best of any in Italy. One peculiarity of it, deserving notice, is the stillness of its atmosphere; high winds being comparatively of rare occurrence. And this quality of calmness is valuable in a winter climate for pulmonary diseases; more especially for diseases of the larynx, trachea and bronchia. It is also of great importance to invalids generally, as it enables them to take exercise in the open air at a much lower temperature, than they could otherwise do. To patients labouring under bronchial irritation, wind is peculiarly hurtful. When wind does occur at Rome, during the winter and spring, it is generally from the north, (tramontana,) at least when it continues for any considerable time. From this quarter there are occasional storms of cold wind; but these are of short duration, being limited, with surprising regularity, to three days. The Tramontana is a dry, keen, and irritating wind, resembling in its effects the cold, sharp winds of Provence; and is equally to be guarded against by invalids; who should not stir out of the house while it blows with much force. The effects of this wind are accurately described by Celsus:—'Aquilo tussim movet, fauces exasperat, ventrem adstringit, urinam supprimit, horrores excitat item dolorem lateris et pectoris. Sanum tamen corpus spissat et mobilius atque expeditius reddit.' The southerly winds during the winter and spring do not produce much inconvenience to invalids at Rome. Even the relaxing and enervating effects of the *Sirocco* are not much felt, except by the more sensitive, and plethoric among the healthy, and by them only after it has continued to blow for a few days. Debilitated invalids, on the other hand, who suffer from great irritability, and a great degree of morbid sensibility of body, commonly feel the winter sirocco pleasant. In its effects on the body this wind is directly opposed to the Tramontana. 'Auster aures hebetat, sensus tardat, capitis dolorem movet, alvum solvit, totum corpus efficit hebes, humidum, languidum.' Notwithstanding the character given of this wind by Celsus, it is the favourite of the modern Romans; and during the prevalence of the winter sirocco they feel the full enjoyment of health. In the months of March and April, winds are more frequent at Rome; they set in generally in the forenoon, and continue till sun-set, when they generally subside, leaving the nights calm and serene; and with a cloudless brilliancy which, at this season, is peculiar to Italy. The effects of these keen spring winds, combined with that of a powerful sun, are severely felt by the sensitive invalid; though, as far as I could observe or learn from the testimony of others, these effects are considerably less in degree than those resulting from the same causes, during this season, at Nice, and perhaps even at Pisa." 111.

We must pass over the malarious and other diseases prevalent at Rome, (because we shall notice them separately) in order to advert to some other points connected with climate as affecting invalids.

### SUMMER RESIDENCE.

This is a matter of great importance to those who resort to Italy or other southern situations for the Winter. To those who have wintered in Italy, two plans present themselves for Summer—either to re-cross the Alps, or to select the most favourable spot on the Italian side. By the first, the invalid will escape the oppressive heat of the Italian Summer—by the latter, he will avoid the inconvenience and expense of a long journey. Dr. C. advises consumptive invalids generally to quit Italy—among consumptive invalids he includes all those who are threatened with phthisis. The heat of Italy will disagree in proportion to the advanced period of the disease. There may be a few exceptions, among those of torpid constitutions, with little nervous excitability, and defective cutaneous secretions. These may more safely remain beyond the

Alps. The spots selected for this purpose are usually, Naples and its vicinity, Sienna, and the Baths of Lucca. These are the most eligible summer residences south of the Appenines. We can only notice the

BATHS OF LUCCA.

This agreeable little watering place is situated among the Appennines behind Lucca, and is much frequented during the Summer, on account of the coolness of the air and the medicinal powers of its waters. The mean temperature of the Summer here is about six degrees higher than the Summer in London. In the middle of the day, however, the heat is very great—the evenings and nights being cool and pleasant.

### SWITZERLAND.

This interesting country presents many conveniences for the invalid; but our author cautions the phthisical patient against the sudden and great vicissitudes experienced among the Alps. Those, however, who are only threatened with consumption may pass the Summer in Switzerland, provided common caution is taken against aerial vicissitudes. They should avoid, he properly observes, those excursions which require great exertions—cause much fatigue—and expose to currents of cold air when the body is heated.

"It will not, I hope, be supposed from any thing now stated, that I wish to throw obstacles in the way of young persons threatened with consumption taking exercise in the open air. This is so far from being my intention, that I think such persons can hardly be too much in the open air. All I wish to inculcate is, that they should be careful not to convert the best of all preventives into a source of evil. For this class of invalids, horse exercise is of all others the most favourable. I am convinced from experience that frequent and gentle motion through a mild atmosphere is one of the most soothing and invigorating measures which we possess, for allaying an irritated and congested state of the mucous membranes of the lungs, and improving the general health." 146.

Of the different parts of Switzerland, Dr. C. prefers the neighbourhood of Geneva. Vevey is hot in July and August—and the higher situations about Lausanne are exposed to the cutting Bise wind.

We must close the climate of Switzerland, and we may say, of Europe, with the following short extract respecting the—

"CURE DE RAISINS."

"The subjects of pulmonary affections, who have spent the summer in Switzerland, will do well to try the '*Cure de Raisins*.' Of the salutary effects of ripe grapes, taken in considerable quantity for some time, there can be no question. In irritation of the mucous membrane of the lungs and digestive organs, and in congestive states of the abdominal viscera, with a disposition to hæmorrhoids, ripe grapes taken, for some weeks, in the quantity of several pounds a day, with a light diet, and abstinence from wine and every thing exciting, will often prove very beneficial." 149.

The first part of the work before us closes with some account of the climate of Madeira, taken chiefly from the writings of Drs. Heineken and Renton, with which we have long ago made our readers acquainted. The second part of the volume, on Diseases of the Chest and Abdomen, will furnish another article in our next number.

It will be evident to our readers, even from the extracts which we have given, that Dr. Clark deserves great praise for the pains, which he has taken in collecting climatorial facts and observations on the various places resorted to by invalids for health. Dr. C.'s writings are characterised more by judgment than by genius—by patient research and careful observation than by originality of thought, and splendour of talent. His language, as our readers must have observed, is as pure as the virgin snow on the summit of MONTE ROSA—and almost as *cold!* Dr. Clark is a philsopher of the first water. He can tread on the ashes of the Cæsars, the senators and the poets of Rome, with as much *sang froid* as on the Macademised pebbles in Hanover Square ! The sight or the recollections of the Tarpeian rock, the chaffing Tiber, the peaceful Tusculum, the tomb of Virgil, or the fiery floods of Vesuvius, never appear to excite an emotion in his mind perturbative of the calm tenor of investigation in which he is engaged :—at all events no emotion is suffered to clothe itself in the garb of language ! So again, our most philosophic author can traverse the horrid steeps of the Grimsel, the terrific precipices of St. Gothard, or the yawning *crevasses* of the Glaciers, in quest of health, or in meditation on climate, with as much composure of mind as he would pace the trottoir of Oxford Street, or ascend the stairs of a patient's bed-chamber. Do we upbraid him with this imperturbable philosophy—

We think the ROMANS call it Stoicism ?

Far from it ! We admire that "singleness of heart," as well as of imagination, which can remain unimpressed and unimpassioned by every surrounding scene or circumstance, save that which forms the immediate subject of inquiry—the useful object of pursuit !

Dr. Clark's work must form an indispensible companion to every invalid who seeks restoration of health or prolongation of life beneath a milder sky than that which lours over his native land. It will form a very useful book of reference for the medical practitioners of this country, few of whom fail to be consulted as to the influence of change of climate over various diseases. But as there is a large class of our brethren who will not, or cannot purchase or peruse new publications, we have taken considerable pains in placing within their reach a great mass of useful climatorial information, British and foreign, in this article. In this part our labour has, of course, been purely analytical. In the succeeding article, on the nature and treatment of various important diseases, we may take the opportunity of offering some comments on certain points of doctrine and practice laid down in the work of the amiable and intelligent author before us. In the mean time we have no hesitation in saying that this volume on Climate has added considerably to the well-earned reputation which Dr. Clark had previously acquired by his private practice and public writings.

